# Safety of propofol sedation administered by interventional radiologists for radiofrequency ablation in patients with hepatocellular carcinoma

**DOI:** 10.1007/s11604-024-01615-2

**Published:** 2024-06-26

**Authors:** Shintaro Kimura, Miyuki Sone, Shunsuke Sugawara, Chihiro Itou, Mizuki Ozawa, Tetsufumi Sato, Yoshiyuki Matsui, Yasuaki Arai, Masahiko Kusumoto

**Affiliations:** 1 Department of Diagnostic Radiology, National Cancer Center Hospital, 5-1-1 Tsukiji, Chuo-ku, Tokyo, 104-0045 Japan; 2 Cancer Medicine, The Jikei University Graduate School of Medicine, Tokyo, Japan; 3 Department of Anesthesia and Intensive Care, National Cancer Center Hospital, Tokyo, Japan; 4 Department of Urology, National Cancer Center Hospital, Tokyo, Japan

**Keywords:** Hepatocellular carcinoma, Radiofrequency ablation, Sedation, Non-anesthesiologist administration of propofol, Interventional radiology

## Abstract

**Purpose:**

To evaluate the safety of propofol sedation administered by interventional radiologists during radiofrequency ablation (RFA) for hepatocellular carcinoma (HCC).

**Materials and methods:**

Propofol sedation was administered by interventional radiologists in 72 patients (85 procedures, 93 tumors) during RFA for HCC between August 2018 and December 2020. Interventional radiologists equipped with adequate knowledge and skills in sedation and respiratory management were responsible for sedation. Sedation was carefully assessed based on vital signs, including end-tidal carbon dioxide, consciousness status, and bispectral index. The primary endpoint was the incidence of sedation-related complications, which were evaluated separately as respiratory and cardiovascular complications. Secondary endpoints were technical success rate, ablation-related complications, and local tumor control rate. Technical success was defined as completion of ablation in the planned area. Complications were evaluated using the Clavien–Dindo classification. Sedation-related complications, technical success rate, and ablation-related complications were evaluated on a procedure basis, and local tumor control was evaluated on a tumor basis.

**Results:**

Respiratory and cardiovascular complications were observed in eight (8/85, 9.4%) and two (2/85, 2.4%) patients, respectively. Four patients required the jaw thrust maneuver due to glossoptosis, whereas a decrease in oxygen saturation to < 90% was recorded in the other four patients. However, these were temporary, and none required manual ventilation or endotracheal intubation. Bradycardia (< 50 bpm) was detected in two patients; one recovered immediately without treatment, whereas the other rapidly improved after atropine sulfate administration. No severe hypotension (< 80 mmHg) was observed. The technical success rate was 100% (85/85). Grade 3 ablation-related complications were identified in three patients (3/85, 3.5%). The local tumor control rate was 95.7% (89/93).

**Conclusion:**

Propofol sedation can be safely administered by interventional radiologists during RFA for HCC. Although it requires special safety considerations, it may be a sedation option during hepatic RFA.

## Introduction

Hepatocellular carcinoma (HCC) is a malignant tumor mainly caused by a hepatic virus infection and is the sixth most common malignant tumor worldwide and the second most deadly [1]. Radiofrequency ablation (RFA) is a curative treatment for early stage HCC and is reported to achieve satisfactory results for local control and post-treatment survival [2–8].

RFA is often accompanied by intense pain and discomfort during treatment. Therefore, appropriate sedation and analgesia are important to reduce pain and ensure safety, which may affect therapeutic efficacy [9–11]. Several options exist for sedation, including the use of benzodiazepines, dexmedetomidine, general anesthesia, and epidural anesthesia, which differ depending on the care settings and conditions at each institution.

Propofol is an intravenous anesthetic agent mainly used for general anesthesia and is also increasingly used as a sedative for relatively painful procedures of short duration with spontaneous breathing, as induction and recovery times are shorter because of its pharmacological properties [12]. Especially in the field of endoscopy, there have been many reports of the efficacy of propofol sedation with regard to recovery time, time to sedation, patient satisfaction, and quality of the examination compared with sedation with benzodiazepines [13–20]. In addition, in recent years, several studies have reported on the efficacy of propofol sedation for hepatic ablation therapy [21–23]. In Japan, benzodiazepine and dexmedetomidine are commonly used sedatives for interventional radiology (IR) procedures and the efficacy of these sedatives has been reported [11, 24]. However, they have longer induction and recovery times compared to propofol, which can be a risk of oversedation after the procedure and a disadvantage in high-volume centers where many procedures need to be performed efficiently in a short period of time. Furthermore, propofol does not require dose adjustment in patients with impaired hepatic function. This is an advantage of propofol sedation during hepatic RFA.

Propofol sedation has a risk of severe adverse events, such as respiratory depression, hypotension, and bradycardia; hence, it should be used correctly and ideally managed by anesthesiologists. The Royal College of Radiologists’ sedation guidelines state that propofol should remain an “anesthesiologist-only” drug [25]. However, depending on the region and institution, a serious shortage of anesthesiologists may make it difficult to manage sedation for treatments outside the operating room. Alessandra et al. reported that more than half of sedation in European IR was performed by non-anesthesiologists, including IR nurses and interventional radiologists [26]. In addition, in Japan, the shortage of anesthesiologists is a serious problem, and most IR is performed without anesthesiologists. To overcome this problem, the efficacy and safety of non-anesthesiologist administration of propofol (NAAP) were demonstrated in the field of endoscopy, and the guidelines about NAAP have been published by the European Society of Gastrointestinal Endoscopy [27]. However, to the best of our knowledge, there are no reports about propofol sedation administered by interventional radiologists. We hypothesized that propofol sedation administered by interventional radiologists equipped with appropriate knowledge and skills in sedation and respiratory management is acceptable and feasible. In this study, we retrospectively evaluated the safety of propofol sedation administered by interventional radiologists during RFA for HCC.

## Materials and methods

### Study design

This single-institute retrospective study was approved by our institutional review board (2023-142), and written informed consent was obtained from each patient prior to any procedure in accordance with the principles embodied in the Helsinki Declaration. Patients who underwent percutaneous RFA for HCC under propofol sedation administered by interventional radiologists between August 2018 and December 2020 were included in this study.

### Patients

A total of 73 patients underwent RFA for HCC between August 2018 and December 2020 at our institution. One patient received conventional sedation with midazolam because of a soy allergy. Following the exclusion of this patient, 72 (85 procedures, 93 tumors) were included in this study.

The inclusion criteria for RFA were as follows: age ≥ 20 years; the presence of histologically confirmed or clinically diagnosed HCC; no more than three intrahepatic tumors and tumor size ≤ 3 cm; the absence of any benefit from treatment with established efficacy (e.g., resection); the presence of Child–Pugh A or B class, American Society of Anesthesiologists physical status (ASA-PS) I, II or III [28]; platelet count ≥ 50,000/mm^3^, and prothrombin time ≤ 1.5 (international normalized ratio).

### Propofol sedation

**Fig. 1:**
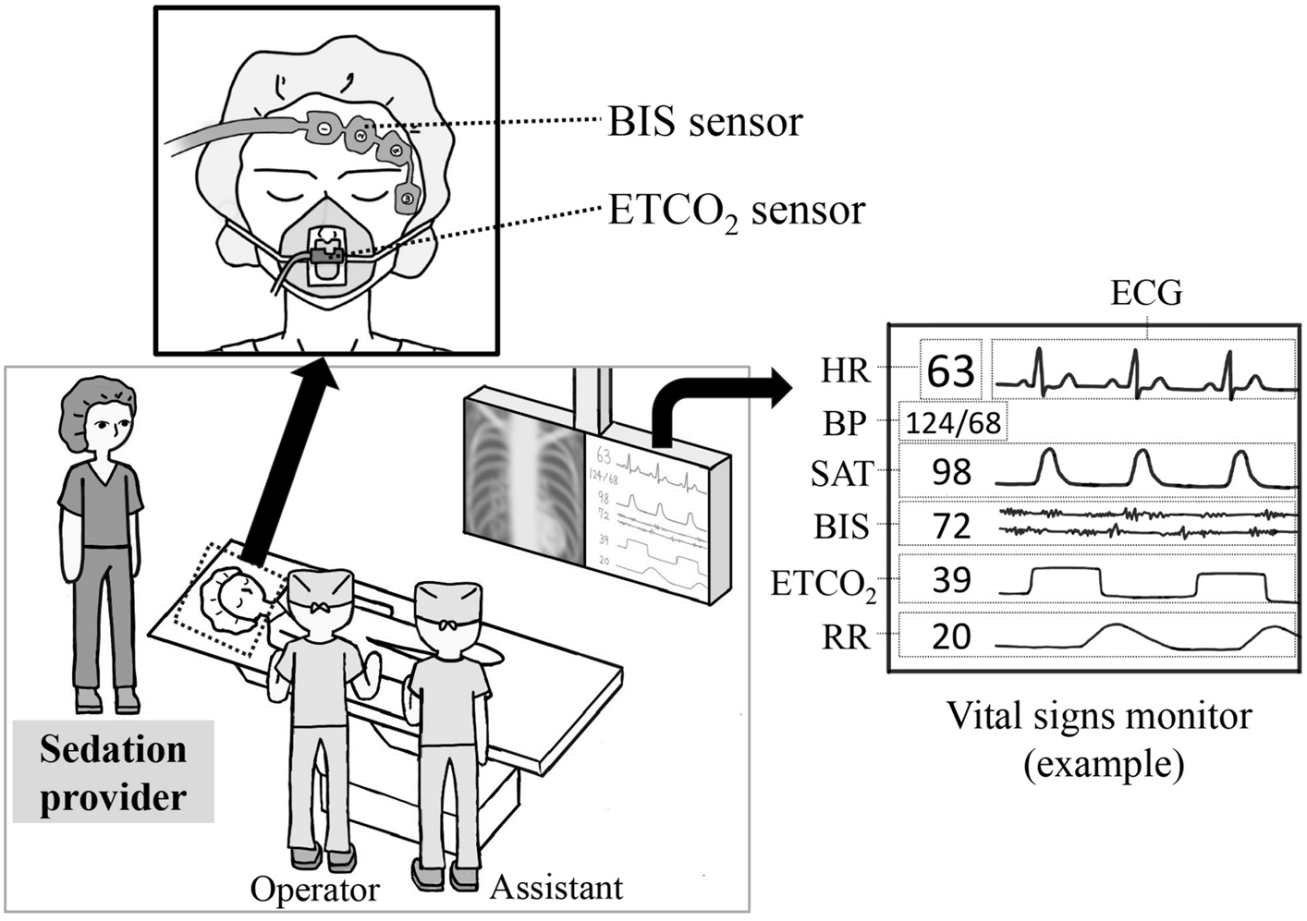
Propofol sedation is administered by a sedation provider, who is not involved in the RFA procedure. The sedation level is assessed with ECG, HR, BP, SAT, BIS, ETCO_2_, and RR, in addition to patients conscious status. The BIS sensor is placed on the forehead of the patient. The face mask is placed on the patient for oxygen supply and ETCO_2_ measurement. *HR* heart rate, *ECG* electrocardiogram, *BP* blood pressure, *SAT* saturation, *BIS* bispectral index, *ETCO*_*2*_ end-tidal carbon dioxide, *RR* respiratory rate

Approval to use propofol sedation in the IR department was obtained from the medical safety department in our institution. Propofol sedation was performed by nine interventional radiologists with a mean of 12 (3–26) years of IR experience. These radiologists had attended a course given by anesthesiologists on the use of propofol and received on-the-job training from anesthesiologists during the first NAAP in the IR department. The NAAP guideline for gastrointestinal endoscopy strongly recommends that sedation providers have previous experience in intensive care medicine or anesthesia to acquire skills in respiratory management, such as airway protection and manual ventilation [27]. All nine interventional radiologists satisfied this criterion. An interventional radiologist who was not involved in the RFA procedure was responsible for the sedation (Fig. 1). The sedation provider adjusted the dose of propofol depending on consciousness level and hemodynamic/respiratory status. In all cases, propofol sedation was combined with fentanyl for analgesia. The administration of propofol and fentanyl was decided based on the protocol developed with the anesthesiologists. Propofol sedation was usually administered just before ablation, since respiratory synchronization was sometimes required for the accurate puncture of the RFA needle or the dissection technique. Therefore, the RFA needle insertion and dissection were performed without propofol.

Propofol was intravenously administered by bolus at 0.5 mg/kg, followed by continuous intravenous infusion at 1.5–2.0 mg/kg/h. Fentanyl was intravenously administered at 0.5 μg/kg initially and 0.2 μg/kg every 30 min thereafter. In cases of patient body movement due to insufficient sedation, the sedation provider added propofol (10–20 mg) and/or fentanyl (10–20 μg) while evaluating the patient’s consciousness status and changes in vital signs. For high-risk patients, such as older adults (≥ 80), patients with an ASA-PS of III, and those with a history of ischemic heart disease or chronic obstructive pulmonary disease, sedation was administered with a 15% reduction of propofol and fentanyl. The risk of airway obstruction in patients with obesity [body mass index (BMI) > 30 kg/m^2^] was evaluated in advance using the STOP-BANG questionnaire, which is a simple screening tool for obstructive sleep apnea [29].

During sedation, percutaneous oxygen saturation, respiratory rate, heart rate, electrocardiogram, and endo-tidal carbon dioxide were monitored continuously, and blood pressure was measured non-invasively every 5 min. Oxygen was administered at 4 L/min from a capnography mask (cap-ONE mask, Nihon Kohden, Tokyo, Japan). Sedation depth was assessed using the Modified Observer’s Assessment of Alertness/Sedation scale every 5 min. A Bispectral Index (BIS) Monitoring System (Medtronic, Minneapolis, MN, USA) was used to objectively evaluate the sedation depth, and the BIS value was set to 60–80 as an appropriate sedation depth (Fig. 1). After the procedure, the patient was moved to the recovery room, and the sedation provider evaluated the vital signs and level of arousal using the modified Aldrete scoring system, which is a commonly used tool to clinically assess the physical status of patients recovering from anesthesia [30]. Patients with a score of 7 or higher were allowed to leave the recovery room and return to the general ward. An on-call anesthetist was available from within the hospital if required.

### Procedure of radiofrequency ablation

Treatment was performed in a room equipped with ultrasound (Aplio i800, Aplio 300, or Nemio XG; Canon Medical Systems, Tochigi, Japan) and angio-computed tomography (CT) (INFX-8000C/Aquillion 16; Canon Medical Systems, Tochigi, Japan), a hybrid system combining CT with X-ray fluoroscopy. A 17-gauge cooled-tip electrode (Cool Tip, Medtronic, Minneapolis, MN, USA) was inserted under image guidance. Ultrasound guidance was used preferentially for lesions visible on ultrasound, and CT fluoroscopy was used for lesions not visible on ultrasound. If lesions were not visible on ultrasound or non-enhanced CT, such as small tumors, isoechoic tumors, and tumors that were difficult to detect because of their location (deep location or near the hepatic dome), transarterial chemoembolization/transarterial embolization using lipiodol (Guerbet, Aulnay-sous-Bois, France) for labeling was carried out on the day before ablation under minimal to moderate sedation without propofol. For lesions under the hepatic dome or near the gastrointestinal tract, dissection was performed using artificial ascites, artificial pleural effusion, or a balloon catheter [31, 32].

### Study endpoints

The primary endpoint was the safety of propofol sedation administered by interventional radiologists, which was evaluated separately for respiratory and cardiovascular complications. Respiratory complications included the following: increasing the oxygen flow because of a decrease in oxygen saturation to < 90%, securing the airway through the jaw thrust maneuver, manual ventilation, and endotracheal intubation. Cardiovascular complications included the following: systolic blood pressure < 80 mmHg and heart rate < 50 bpm. The secondary endpoints were the technical success rate, ablation-related complications, and local tumor control rate. Technical success was defined as completion of ablation in the planned area. Complications were evaluated using the Clavien–Dindo classification [33]. Local recurrence was defined as the appearance of an enhanced area in contact with the ablated zone, which was evaluated using dynamic CT or magnetic resonance imaging every 3–6 months after RFA. Sedation-related complications, technical success rate, and ablation-related complications were evaluated on a procedure basis, and local tumor control was evaluated on a tumor basis.

### Statistical analysis

Descriptive statistics are presented as mean ± standard deviation for variables with normal distribution and median (minimum–maximum) for variables with non-normal distribution. Qualitative variables are expressed as frequencies and percentages.

## Results

### Characteristics of patients and tumors

The patient characteristics are summarized in Table 1. Seventy-two patients (85 procedures, 93 tumors) were included in this study. The median patient age was 73 (51–90) years, and 64 were men. The median BMI was 23.7 (19.1–32.5) kg/m^2^, and the ASA-PS was 2/3 = 61/11. The median tumor diameter was 16 (7–31) mm, and the number of tumors per session was 1 in 77 cases and 2 in 8 cases.

**Table 1:** Baseline characteristics of the patients and tumors

Variable	*n* = 72 patients
Age (years), median (range)	73 (51−90)
Sex, male:female	64:8
BMI (kg/m^2^), median (range)	23.7 (19.1−32.5)
ASA physical status, *n* (%)	
2	61 (85)
3	11 (15)
Child–Pugh class, *n* (%)	
A	70 (97)
B	2 (3)
Viral infection, *n* (%)	
HBsAg positive	21 (29)
Anti-HCV positive	24 (33)
Both positive	1 (1.4)
Number of lesions treated per session^a^, 1:2	77:8
Tumor diameter (mm), median (range)^b^	16 (7−31)

*BMI* body mass index, *HBsAg* hepatitis B surface antigen, *HCV* hepatitis C virus, *ASA* American Society of Anesthesiologists

^a^A total of 85 procedures

^b^A total of 93 tumors

### Safety of propofol sedation

Sedation details and sedation-related complications are presented in Table 2. The median sedation time with propofol was 22 (9–154) min, and the median dosage of propofol was 4.3 (1.3–14.2) mg/kg/h. The median dosage of fentanyl used as an analgesic was 0.80 (0.17–2.55) μg/kg/h.

**Table 2:** Sedative details and sedation-related complications

Variable	*n* = 85 procedures
Propofol dosage (mg/kg/h), median (range)	4.3 (1.3–14.2)
Sedation time with propofol (min), median (range)	22 (9–154)
Fentanyl dosage (μg/kg/h), median (range)	0.80 (0.17–2.55)
Respiratory complications, *n* (%)	8 (9.4)
Glossoptosis	4 (4.7)
Decrease in oxygen saturation < 90%	4 (4.7)
Cardiovascular complications, *n* (%)	2 (2.4)
Systolic blood pressure < 80 mmHg	0 (0)
Heart rate < 50 bpm	2 (2.4)

Respiratory complications were observed in eight patients (8/85 procedures, 9.4%). The jaw thrust maneuver was performed in four patients due to glossoptosis, and increasing the oxygen flow was required in the other four patients because of a decrease in oxygen saturation to < 90%. All these were temporary, and no cases required manual ventilation or endotracheal intubation.

Cardiovascular complications were observed in two patients (2/85 procedures, 2.4%). Bradycardia (< 50 bpm) was observed in two patients; one improved without treatment, whereas the other rapidly improved after atropine sulfate administration. No cases with systolic blood pressure < 80 mmHg were observed.

### Efficacy of propofol sedation

The technical success rate was 100% (85/85 procedures). Severe body movement, which forced the reduction of the generator power, was not observed during the procedure.

Three cases of grade 3 ablation-related complications were observed: a case of liver abscess, a case of intra-abdominal abscess, and a case of pneumothorax. All these improved after percutaneous drainage.

Local recurrence was observed in 4 out of 93 lesions, and the local tumor control rate was 95.7% (89/93) with a median follow-up period of 9 months (Table 3).

**Table 3:** Procedural details and ablation-related complications

Variable	*n* = 85 procedures
Procedure time (min), median (range)	65 (30–260)
Total duration of ablation per tumor (min), median (range)	11 (8–21)
TAE/TACE before RFA, *n* (%)	52 (61)
Local tumor control rate, (%)^a^	95.7
Ablation-related complications (≥ Grade 3), *n* (%)	
Liver abscess (Grade 3)	1 (1.2)
Intra-abdominal abscess (Grade 3)	1 (1.2)
Pneumothorax (Grade 3)	1 (1.2)

*TAE* transarterial embolization, *TACE* transarterial chemoembolization, *RFA* radiofrequency ablation

^a^Local tumor control was evaluated on a tumor basis (total: 93 tumors)

## Discussion

During RFA for HCC with propofol sedation administered by interventional radiologists, respiratory and cardiovascular complications were observed in 9.4% and 2.3% of patients, respectively. All these were grade 2 or lower complications, and no serious sedation-related complications were observed in this study. Furthermore, with respect to efficacy, the technical success rate was 100%, and the local tumor control rate was 95.7%.

Appropriate sedation is important to not only prevent adverse events but also improve patient satisfaction and oncological outcomes. In the field of endoscopy, the superiority of propofol sedation over benzodiazepine was presented by randomized controlled trials and meta-analyses [13–20]. Regarding hepatic ablation therapy, a retrospective study about sedation outcomes during microwave ablation for liver tumors showed that midazolam sedation was associated with significantly more pain during treatment and a significantly higher local recurrence rate than propofol sedation and general anesthesia [21]. The patient’s pain was affected by the power of the generator [11], which changed during the ablation. After so-called “break” (i.e., impedance increase by 30 Ω or 30% compared with baseline value), the generator output was stopped, and patient’s pain was rapidly relieved; therefore, long-acting sedative agents may cause oversedation after the break. Propofol is a short-acting sedative agent and may provide appropriate sedation management for RFA. In addition, propofol does not need dose adjustment in patients with impaired hepatic function as opposed to benzodiazepine, including midazolam [34, 35]. This is advantageous for treating HCC development in patients with chronic liver disease.

Propofol sedation is ideally managed by anesthesiologists; however, in areas where anesthesiologists are in short supply, procedural sedation is compelled to be administered by non-anesthesiologists. NAAP is one of the options to overcome this problem. In the field of endoscopy, with appropriate training and monitoring devices, the safety of NAAP has already been demonstrated. A meta-analysis of 26 prospective observational studies showed that the safety of propofol sedation administered by endoscopists was associated with similar safety compared with anesthesia provider-administered propofol sedation [36]. In addition, the safety of propofol sedation administered by cardiologists during atrial fibrillation ablation was already reported [37, 38]. This is the first study to evaluate the safety of propofol sedation administered by interventional radiologists who have acquired knowledge and skills of sedation and respiratory management, and it can be safely performed by a rigorous assessment of respiratory/hemodynamic status and sedation depth with appropriate monitoring devices.

Capnography provides continuous dynamic assessment of the ventilatory status of patients, and its use was recommended by several sedation guidelines [27, 39, 40]. The lag times from the start of apnea to capnographic findings and the development of hypoxemia are approximately 5 s and another 10–20 s, respectively [41]; thus, capnography can provide an extremely early warning of a ventilation disorder. RFA is usually performed in the supine position, which is more likely to cause a ventilation disorder due to glossoptosis than other endoscopic procedures usually performed in the lateral or prone position. Because glossoptosis under propofol sedation appears earlier than respiratory depression, ventilatory assessment by merely observing chest wall movements may delay the detection of ventilatory impairment. The use of capnography is particularly useful in these cases. Furthermore, the BIS monitoring system is effective as a minimally invasive and objective indicator of sedation depth; it was reported to reduce the risk of oversedation, allow a reduced dose of propofol, and shorten the duration of recovery time in the field of endoscopy [42, 43]. In our study, these monitoring devices prevented severe respiratory depression, hypotension, and bradycardia by promptly detecting the signs of oversedation.

It is also crucial for the person in charge to be focused only on sedation. According to several guidelines, sedation should be administered by a physician who does not participate in the procedure [26, 39, 40]. To ensure safety, continuous patient monitoring by the sedation provider is essential.

Moreover, the appropriate use of analgesics is also important for the maintenance of ideal sedation. Propofol is considered to have no analgesic properties and must, therefore, be used in combination with appropriate analgesics. Fentanyl has unique pharmacological properties, including rapid serum clearance, high potency, and minimal histamine release. Additionally, unlike pentazocine, fentanyl has no ceiling effect, which is a phenomenon in pharmacology and medicine where the maximum therapeutic effect of a drug is reached at a specific dose, and increasing the dose does not result in further improvement. These properties are suitable for RFA, which is often accompanied by intense pain with a short duration. In our study, no severe body movement was observed during ablation, and satisfactory rates regarding local tumor control and complications were obtained, thereby suggesting the efficacy of propofol and fentanyl in RFA for HCC.

The present study had some limitations. First, this study had no control group and could not be compared with other sedative methods. Second, this was a retrospective study with small population conducted at a single center. The safety and efficacy of propofol sedation may depend on the knowledge and experience of the interventional radiologist as a sedation provider. Our results might not reflect those of other institutions with limited experience in sedation or those of interventional radiologists with inadequate training in respiratory management. Therefore, a multicenter study with standardized education and training systems about sedation would be necessary to extrapolate the results of our study. Third, patients’ pain and satisfaction were not evaluated as endpoints. Patient-reported outcomes are important factors in assessing the efficacy of sedation. Thus, a prospective study including patient-reported outcomes is required. Fourth, in some countries, including Japan, the use of propofol for the procedural sedation is not approved. In these countries, off-label use of propofol must be approved by an institutional review board or medical safety department.

In conclusion, propofol sedation can be safely administered by interventional radiologists during RFA for HCC. Although it requires special considerations for safety management, it may be a sedation option during hepatic RFA.
